# Detection of cardiac apoptosis by [^18^F]ML-10 in a mouse model of permanent LAD ligation

**DOI:** 10.1007/s11307-022-01718-0

**Published:** 2022-03-29

**Authors:** Maximilian Fischer, Jessica Olivier, Simon Lindner, Mathias J. Zacherl, Steffen Massberg, Peter Bartenstein, Sibylle Ziegler, Matthias Brendel, Sebastian Lehner, Guido Boening, Andrei Todica

**Affiliations:** 1 Medizinische Klinik und Poliklinik I, Klinikum der Universität München, Ludwig-Maximilians-Universität, Marchioninistrasse 15, 81377 Munich, Germany; 2 DZHK (German Centre for Cardiovascular Research), Partner Site Munich Heart Alliance, 80802 Munich, Germany; 3 Department of Nuclear Medicine, University Hospital, LMU Munich, Marchioninistr. 15, 81377 Munich, Germany; 4 Ambulatory Healthcare Center Dr. Neumaier & Colleagues, Radiology, Nuclear Medicine, Radiation Therapy, Regensburg, Germany; 5 DIE RADIOLOGIE, Munich, Germany

**Keywords:** Cardiac positron emission tomography, Autoradiography, [^18^F]ML-10, [^18^F]FDG, Apoptosis, Myocardial infarct

## Abstract

**Purpose:**

The loss of viable cardiac cells and cell death by myocardial infarction (MI) is still a significant obstacle in preventing deteriorating heart failure. Imaging of apoptosis, a defined cascade to cell death, could identify areas at risk.

**Procedures:**

Using 2-(5-[^18^F]fluoropentyl)-2-methyl-malonic acid ([^18^F]ML-10) in autoradiography and positron emission tomography (PET) visualized apoptosis in murine hearts after permanent ligation of the left anterior descending artery (LAD) inducing myocardial infarction (MI). 2-deoxy-2-[^18^F]fluoro-D-glucose ([^18^F]FDG) PET imaging localized the infarct area after MI. Histology by terminal deoxynucleotidyl transferase dUTP nick end labeling (TUNEL) staining validated apoptosis in the heart.

**Results:**

Accumulation of [^18^F]ML-10 was evident in the infarct area after permanent ligation of the LAD in autoradiography and PET imaging. Detection of apoptosis by [^18^F]ML-10 is in line with the defect visualized by [^18^F]FDG and the histological approach.

**Conclusion:**

[^18^F]ML-10 could be a suitable tracer for apoptosis imaging in a mouse model of permanent LAD ligation.

**Supplementary Information:**

The online version contains supplementary material available at 10.1007/s11307-022-01718-0.

## Background

Apoptosis and its intracellular mechanism have already been described in numerous reviews [1–3]. Either the intrinsic or the extrinsic pathway initiates the apoptosis cascade. Stress signals in the endoplasmatic reticulum, oxidative damage, ischemia, and DNA damage trigger the release of cytochrome c from the mitochondrial membrane. The stressor-induced imbalance of pro- and anti-apoptotic signals and lead to the formation of Apaf-1 protein. Cleavage of the caspase-9 starts a cascade resulting in the activation of so-called executioner caspases and ultimately programmed cell death [4].

The extrinsic apoptosis pathway, in contrast, is activated by death receptors on the cell membrane. Binding of death ligands such as Fas ligand (FasL) or tumor necrosis factor α (TNFα), a complex called death-inducing signaling complex (DISC), is formed. DISC further induces by initiator caspases the activation of executioner caspases. At this point, both pathways converge in execution.

This phase is represented by biochemical and morphological alteration to the cell in apoptosis (e.g., chromatin condensation, shrinkage, and breakdown of proteins and DNA [5, 6]). The main characteristic at this stage is the ATP-dependent and enzyme-mediated externalization of phospholipid phosphatidylserine toward the outer cell membrane [2]. *In vivo* imaging of apoptosis by positron emission tomography (PET) offers a promising solution and could directly evaluate therapeutic benefits.

The prevalence and mortality due to myocardial infarction (MI) present a significant issue in the western world [7, 8]. In myocardial infarction, the loss of viable heart tissue can ultimately lead to severe heart failure, compromising life span and quality [9]. Cell death, represented by apoptosis, necrosis, and autophagy, occur in cardiac myocytes, fibroblasts, and endothelial cells after MI. Pharmacological and genetic approaches aim to modulate these dynamic processes [10].

The endogenous protein annexin V (~ 36 kDa) is a member of a superfamily and is expressed in many eukaryotic organisms, including humans (reviewed in [11]). Its main advantage is the selective calcium ion concentration-dependent binding and high affinity to phospholipid phosphatidylserine, but not exclusively in apoptotic cells. Despite tremendous research on the development of a clinically feasible radiolabeled annexin V, there are limitations concerning the complex radiolabeling, biodistribution and random uptake in the liver and kidney, slow clearance, interference with calcium ion concentrations, insufficient differentiation of apoptosis, and necrosis [12] and poor tissue penetration [11].

Nevertheless, *in vivo* imaging of apoptosis in myocardial infarction could display a pivotal cardiac diagnostic progress and monitor new therapeutic strategies [13–16].

Another promising small molecule probe is 2-(5-fluoropentyl)-2-methyl-malonic acid (ML-10) (206 Da), which is incorporated and accumulates in apoptotic cells, not in viable or necrotic cells, and thereby could provide an* in vivo* imaging that discriminates those different pathological states [17]. [^18^F]ML-10 was already used to visualize apoptotic cancer cells [18] and assesses the effect of whole-brain radiation therapy on brain cancer and metastasis [19, 20].

In the vascular system, after murine stroke, induced by ligation of the arteria cerebri, [^18^F]ML-10 was detected in the ischemic area, while the accumulation in healthy tissue was absent [21]. Regarding the cardiovascular system, [^18^F]ML-10 accumulation detected apoptotic cells with atherosclerosis-like lesions in rabbits [22]. Ex vivo autoradiography and correlation to histology identified the [^18^F]ML-10 accumulation in the injured aortas. However, published data on the heart are limited.

In this study, cardiac PET imaging of [^18^F]ML-10 and [^18^F]FDG after permanent ligation of the left anterior descending (LAD) artery, combined with histology, assess characteristics of [^18^F]ML-10 to detect and quantify cardiac apoptosis in mice.

## Methods

### Animals

Male C57BL/6 N mice were purchased from Charles River (Sulzfeld, Germany). Animal care and all experimental procedures were performed according to the Guideline for the Care and Use of Laboratory Animals published by the US National Institutes of Health (NIH publication no. 85–23, revised 1996). The mean animal weight was 23.48 ± 1.44 g.

As described previously, the acute myocardial injury was induced in 10-week-old C57BL/6 N mice by permanent surgical ligation of the left anterior descending artery [23, 24]. In brief, after inducing anesthesia by intraperitoneal injection of medetomidine 0.5 mg/kg, midazolam 5.0 mg/kg, and fentanyl 0.05 mg/kg. The animal was placed in a supine position with paws taped to the operation table. A midline cervical skin incision was made along with the reflection of muscles overlying the trachea to allow visualization of the endotracheal tube. After surgical preparation, the mice were intubated with a 19 gauge tube and ventilated at a volume of 0.15 ml at a frequency of 110/min (Mini Vent T845, Hugo Sachs Elektronik, Hegstetten). After tube placement, the cervical skin was sutured (5–0 Ethibond). The mice lay on a heating mat, and body temperature was closely monitored by a rectal probe. Next, the third intercostal space on the left was used to facilitate thoracotomy.

After surgical preparation, the left anterior descending artery (LAD) was ligated by an 8–0 prolene suture resulting in an infarct area of the left ventricle. The diminished blood flow distal to the ligation site was used as intraoperative criteria for successful ligation. In sham control mice, the same procedure except for the LAD ligation was performed. The chest wall was then closed by a 6–0 Ethibond suture of the chest muscles and the skin. Post-analgetic was applied (Buprenovent 0.3 mg/ml) subcutaneously. Anesthesia was reversed by 2.5 mg/kg atipamezole and 0.5 mg/kg flumazenil. The animal was removed from the respirator, the endotracheal tube was withdrawn, and the animal was kept warm on the heating mat till transfer into cages after recovery from anesthesia.

Study protocols complied with the institution’s guidelines and were approved by the Government’s animal ethics committee (ROB-55.2Vet-2532.Vet_02-15–241).

### Radiolabeling procedure of [^18^F]ML-10

Automated production of [^18^F]ML-10 was performed on a Raytest® SynChrom R&D single reactor synthesizer. The solvent containers (SC) were charged with reagents, and cartridges were assembled on the synthesizer. The manufacturing process was performed automatically using the Raytest® control software. No-carrier-added [^18^F]fluoride was produced via ^18^O(p, n)^18^F reaction by proton irradiation of ^18^O-enriched water and directly delivered to a preconditioned ion exchange cartridge (Chromabond PS-HCO_3_-, Macherey Nagel, Trap 1). The trapped [^18^F]fluoride was eluted into the reactor using a mixture of Kryptofix®222 (12.5 mg), potassium carbonate (12.5 µl, 1 M), water (187.5 µl), and acetonitrile (800 µl) from SC 2. The solution was evaporated to dryness by azeotropic distillation. The drying process was repeated after addition of acetonitrile (0.8 ml) from SC 3. The precursor (4 mg) in acetonitrile (0.7 ml) was transferred from SC 1 into the reactor, and the mixture was heated at 125 °C for 10 min. HCl (0.5 ml, 3 M) was added from SC 5 and stirred for 5 min at 125 °C. The reaction mixture was quenched with H_2_O (3.8 ml) from SC 4 and purified via semi-preparative HPLC (Inertsil ODS-4 C18 column, 250 × 10 mm, 5 µm; isocratic elution with 70% (v/v) H_2_O + 0.1% (v/v) H_3_PO_4_ (85 wt. % in water) / 30% acetonitrile; flow: 5 ml/min; UV detection: 254 nm). The HPLC purified product peak was collected in SC 11, diluted with 10 ml H_2_O + 0.1% (v/v) H_3_PO_4_ (85 wt. % in water) from SC 8, and passed over a preconditioned tC18 SepPak Plus Short cartridge (Waters, Trap 3). The cartridge was rinsed with 1 ml H_2_O + 0.1% (v/v) H_3_PO_4_ (85 wt. % in water), and the radiolabelled product was eluted with anhydrous ethanol (1 ml) from SC 7 into the product vial, diluted with saline (9 ml) from SC 9 and filtered through a sterile filter (Cathivex-GV, 0,22 µm, Merck Millipore). RCY 22 ± 6% (*n* = 15) d. c., synthesis time 90 min, RCP > 99%.

The purity was confirmed via analytical HPLC (Phenomenex Gemini C18 column, 250 × 4.6 mm, 5 µm; isocratic elution with 60% H_2_O + 0.1% (v/v) TFA / 40% acetonitrile + 0.1% (v/v) TFA; flow: 1 ml/min; UV detection: 254 nm).

### Autoradiography

Intravenous injection of the tracer [^18^F]ML-10 (~ 16 MBq) was performed 135 min before sacrificing and harvesting the heart. The heart was perfused with 0.9% sodium chloride solution and stored as cryosections at -80 °C. Sections of the heart of 30 µm from the basis to the apex were cut at the cryotome. The heart sections were put into the imaging plate, and after 12 h, the heart sections were analyzed via HD-CR 35 NDT Image plate scanner. Quantification of the images was performed using Advanced Image Data Analyzer (AIDA). The non-infarct area (remote area) above the surgical LAD ligation was used as background estimation. The circular region of interest (ROI) was applied on each heart section for the infarct and remote area at the midventricular section of the heart.

### *In vivo* cardiac PET imaging

[^18^F]PET scans were performed at 2, 4, 6, 24, and 48 h using a dedicated small-animal PET scanner (Inveon Dedicated PET, Preclinical Solutions, Siemens Healthcare Molecular Imaging, Knoxville, TN, USA) as described previously [25, 26].

The animals had free access to food and water until before the scan, as described previously [25–28]. Anesthesia was induced (2.5%) and maintained (1.5%) with isoflurane delivered in pure oxygen at a rate of 1.5 L/min after intubation and mechanical ventilation. The core body temperature was maintained within the normal range using a heating pad and monitored by a rectal thermometer.

After placing an intravenous catheter into a tail vein, approximately 16 MBq of [^18^F]ML-10 was injected in a volume of ~ 150 µl. The catheter was then flushed with 50 µl of saline solution.

The isoflurane narcosis was interrupted for the time of [^18^F]ML-10 tracer uptake.

At the end of the tracer uptake phase of 2 h, the animals were re-anesthetized with isoflurane and placed in a prone position within the PET tomograph. A three-dimensional PET recording was obtained in list mode for 30 min after [^18^F]ML-10 injection. For attenuation and scatter correction, a 7-min transmission scan was performed with a rotating Co-57 source, followed by 30-min emission.

After the [^18^F]ML-10 scan, appr. 20 MBq of [^18^F]FDG in a volume of ~ 100 µl was injected intravenously in the tail vein. The time between the tracer injection was 2.5 h. The catheter was again flushed with 50 µl of saline solution. After 13 min, the transmission was again assessed for 7 min, followed by the emission assessment for 20 min. Mice were sacrificed after the [^18^F]FDG PET scan as approved by the authorities. The recorded data were processed with the Inveon Acquisition Workplace (Siemens Medical Solutions, Knoxville, TN, USA). FDG list-mode acquisitions were reconstructed, as described previously [28]. Reconstructions for [^18^F]FDG and [^18^F]ML-10 was performed using an MAP OSEM 3D algorithm in a 256 × 256 × 159 matrix and dimension of 0.39 × 0.39 × 0.8 mm3. Data were reconstructed as a static image, normalized, corrected for randoms, dead time, decay, attenuation, and scatter.

### PET image analysis

Analysis of PET images was performed by the Inveon Research Workplace (Siemens Medical Solutions) described previously [29, 30].

Inveon Research Workplace was used for assessing, cutting, and fusion of the [^18^F]ML-10 and [^18^F]FDG PET images. A cubic volume of interest (VOI) was used to evaluate the infarct area, which was confirmed by the absence of [^18^F]FDG uptake. Correct VOI placement was verified in three projections (axial, sagittal, and coronal). The maximum injected dose per gram (% ID/g)_max_ was determined as the quotient of maximum uptake per ROI (Bq/mL) to injected dose/activity in Bq multiplied by 100. The density of the tissue was set as 1 g/ml.

### Histology and TUNEL Assay

After the PET scans, hearts were excised. After fixation in 4% phosphate-buffered formalin, hearts were cut into 4-µm-thick sections and embedded in paraffin. Standard histological procedure (Haematoxyline-Eosine and Giemsa staining) was performed. According to the manufacturer's protocol, apoptotic cells were stained using ApopTag® Peroxidase *In Situ* Apoptosis Detection Kit S7100 (Millipore) and evaluated by the Axio Version SE64 (Version 4.9) software.

### Statistical analysis

All results were expressed as means with standard deviation. Statistical analysis was performed with Prism (Version 9, GraphPad Software, LLC., San Diego, California).

One-way ANOVA analysis with Tukey’s multiple comparisons was used. The Shapiro–Wilk test was used to test for normal distribution. For groups without normal distribution, the Wilcoxon signed-rank or the Mann–Whitney U test was applied. The differences were considered statistically significant at a P-value of 0.05.

## Results

### Autoradiographic detection of [^18^F]ML-10 in the heart after permanent LAD ligation

**Fig. 1:**
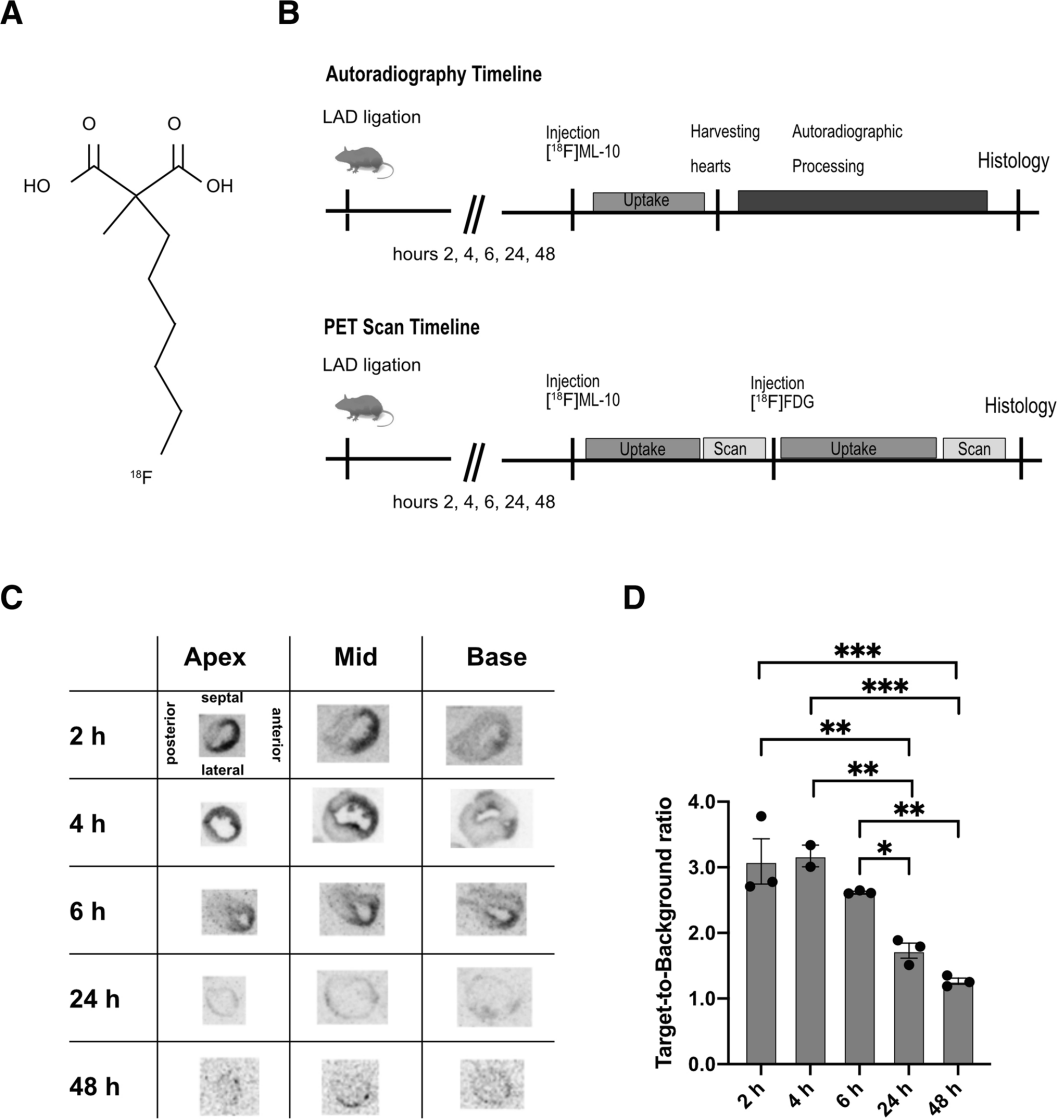
Autoradiography of [^18^F]ML-10 after MI (**A**) Structure of [^18^F]ML-10. (**B**) Schematic study design illustrating the induction of myocardial infarct (MI) by permanent LAD ligation, injection of [^18^F]ML-10 and [^18^F]FDG, autoradiography, PET imaging, and histology at different time points. (**C**) Autoradiographic evaluation of the [^18^F]ML-10 uptake after MI at different time points. Hearts are illustrated at different sections (apex, mid-ventricular, base). (**D**) Quantification of the target-to-background ratio (TBR) after different time points. *N* = 2–3. Data represent mean ± SEM. * *p* < 0.05, ** *p* < 0.01, *** *p* < 0.001

To determine the uptake of [^18^F]ML-10 after permanent LAD ligation, the cardiac accumulation of [^18^F]ML-10 was assessed in the course of 2, 4, 6, 24, and 48 h after MI by autoradiography. The experimental timeline is illustrated in Fig. 1. Hearts were harvested at the indicated time points after MI and cut at different levels for comparing the apical, mid-ventricular, and base of the mouse heart. After permanent LAD ligation, the apical and mid-ventricular sections, as suggested, showed prominent infarcts (Fig. 1C).

To compare the accumulation of [^18^F]ML-10 by autoradiography, the target-to-background ratio was used. [^18^F]ML-10 accumulated early at the peak of 2 to 4 h and then stepwise declined in the course (2 h vs 24 h, *p* = 0.003, 2 h vs 48 h, *p* < 0.001, Fig. 1D). Our observations indicate that [^18^F]ML-10 accumulates in the heart after MI and demonstrates a dynamic process.

### Defect and apoptosis localization and estimation by small-animal PET imaging

We further assessed whether the proposed apoptosis marker provides the feasibility of *in vivo* monitoring the apoptotic processes in the mouse heart.

Therefore, mice were injected with [^18^F]ML-10 and [^18^F]FDG for the identification of cardiac injury.

**Fig. 2:**
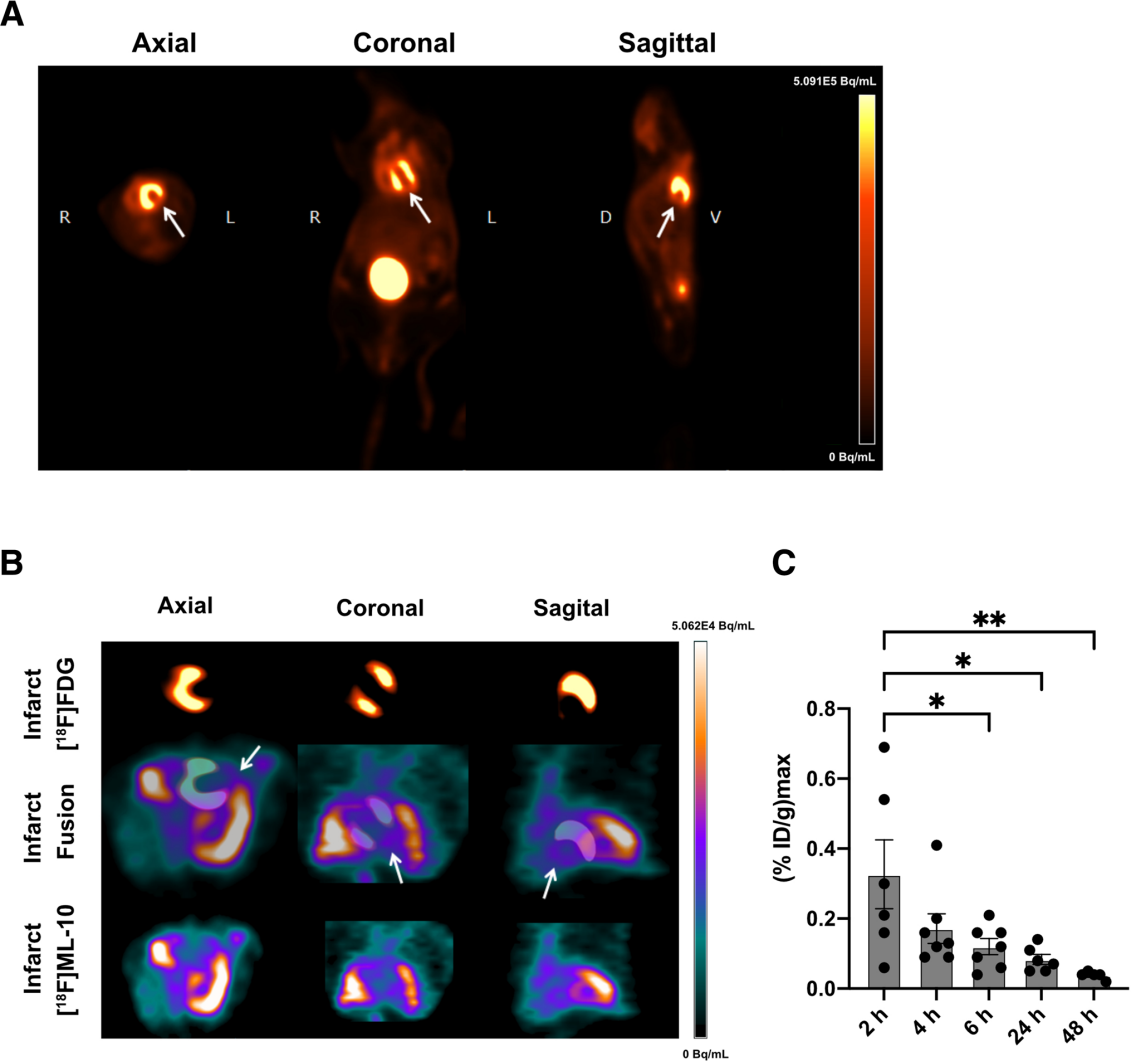
[^18^F]FDG and [^18^F]ML-10 PET imaging after MI**. **(**A**) Representative [^18^F]FDG image illustrating the MI. Arrows indicate the infarct area of the left ventricle. R right, L left, D dorsal, V ventral. Color scale: Volcano. (**B**) Representative images of [^18^F]FDG and [^18^F]ML-10 after permanent LAD ligation. Arrows indicate infarct area detected by diminished [^18^F]FDG uptake and evident [^18^F]ML-10 uptake. Color scale: Ocean. (**C**) Quantification of the injected dose per gram of [^18^F]ML-10 after different time points. *N* = 5–7. Data represent mean ± SEM. * *p* < 0.05, ** *p* < 0.01, *** *p* < 0.001

In the [^18^F]FDG PET imaging, the infarct area indicated by the absent [^18^F]FDG uptake could be identified (Fig. 2A). Additionally, *in vivo* [^18^F]ML-10 PET imaging was performed at different time points, and the two PET imaging modalities were fused to evaluate the [^18^F]ML-10 uptake in the infarct area (Fig. 2B). Of note, as described previously [31], [^18^F]ML-10 also accumulated in the chest and lung after thoracotomy. Here, we could detect a kinetic decrease with a peak maximum of the cardiac uptake of [^18^F]ML-10 after 2 h and a dynamic decrease after MI (2 h vs 24 h, *p* = 0.016, 2 h vs 48 h, *p* = 0.006, Fig. 2C).

### Detection of apoptosis by TUNEL staining after MI

A gold standard of apoptosis detection is the use of TUNEL labeling by histology.

**Fig. 3:**
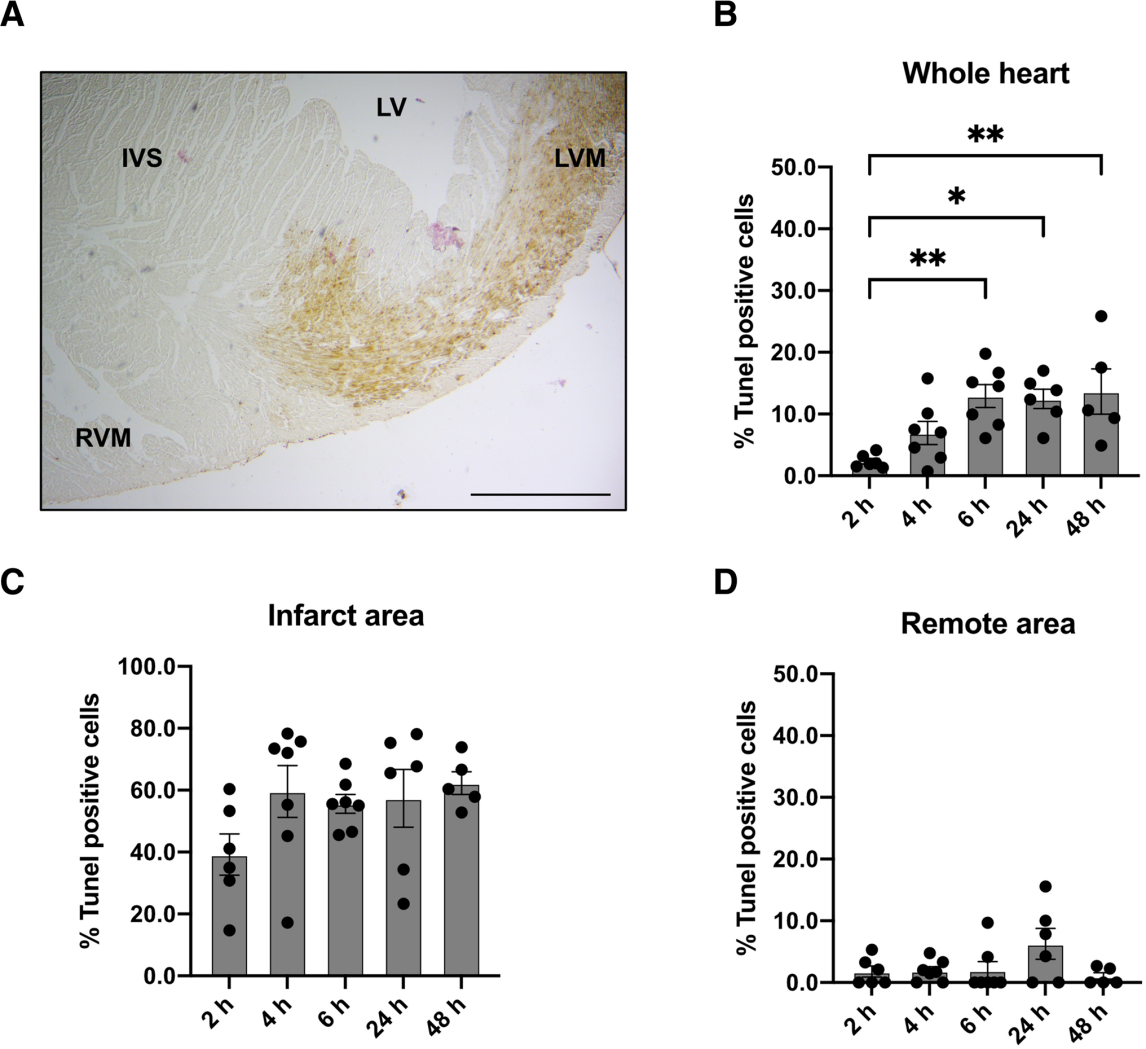
Histological evaluation by TUNEL staining after MI. (**A**) TUNEL staining of the heart after MI. Right ventricular myocardium (RVM), interventricular septum (IVS), left ventricle LV, left ventricular myocardium (LVM). Bar equals 500 μm. (**B**) Quantification of TUNEL positive cells in the whole heart after different time points. *N* = 5–7. (**C**) Quantification of TUNEL positive cells in the infarct area after different time points. *N* = 5–7. (**D**) Quantification of TUNEL positive cells in the remote area after different time points. *N* = 5–7. Data represent mean ± SEM. * *p* < 0.05, ** *p* < 0.01, *** *p* < 0.001

Underlining the apoptotic response after MI, we performed histological analyses of the heart (Fig. 3A). Regarding the whole heart, we could detect a steady increase in the percentage of TUNEL positive cells from 2 to 6 h (2 h vs 6 h, *p* = 0.006, 2 h vs 24 h, *p* = 0.01, 2 h vs 48 h, *p* = 0.007, Fig. 3B). Focusing on the infarct area and the heart's remote area, we observed, as expected, a higher count of apoptotic cells in the infarct area compared to the remote area, where no relevant apoptotic cells could be detected (Fig. 3C and D).

## Discussion

This study is the first to determine the feasibility of the apoptotic marker [^18^F]ML-10 in a mouse model of myocardial infarction. Using different imaging modalities, e.g., autoradiography, small-animal PET, and histology by TUNEL staining, we assessed [^18^F]ML-10 to detect apoptosis in the heart.

Previous studies described the feasibility of [^18^F]ML-10 for apoptosis detection in rats after MI [31]. [^18^F]ML-10 was detected in the early phase after MI at days 1 till 3, while it was not notable at days 5 and 7 after LAD ligation. Regarding data from human cardiac tissue after myocardial infarction, apoptosis could also be detected relatively early, from 2 to 3 h after injury [32]. In addition to published work, our data underline the dynamic process of apoptosis in the first 24 h. After LAD ligation in mice, the peak value of the target-to-background ratio (TBR) in the cardiac autoradiography culminated at 2 to 4 h. Regarding the course of our experiments, the TBR declined over time. Therefore, an early time frame for apoptosis imaging after MI could provide a promising window to quantify [^18^F]ML-10 detection of apoptosis.

The small-animal [^18^F]FDG PET imaging was used to localize the defect after MI [33]. We used this imaging modality to compare the localization of [^18^F]FDG and [^18^F]ML-10. Importantly, in sham operated control mice (see supplement figure S1), no relevant uptake of [^18^F]ML-10 could be detected in PET imaging of the hearts. As a result, we could identify the infarct area by absent [^18^F]FDG and accumulation of [^18^F]ML-10 in the cardiac tissue supplied by the LAD. Using (%ID/g)_max_ detected a steady decline in [^18^F]ML-10 at the observation time frame after MI, which is in line with the decline of TBR in autoradiography. However, the accumulation of [^18^F]ML-10 was not restricted to apoptosis in the heart. Previous publications of rat myocardial infarct also observed the accumulation in the chest wound after thoracotomy [31]. Therefore, the surgical LAD ligation, oropharyngeal intubation, and mechanical ventilation are limitations of the mouse infarct model itself. The extracardiac accumulation might be further different in operating mice and rats.

In addition to the autoradiographic validation, we histologically assessed the heart section for apoptosis by TUNEL staining, which is still the most prominent approach to assessing cardiac cell apoptosis [34]. Of note, we also evaluated the staining for active caspase-3, demonstrating another indicator of early apoptosis. We, however, could not detect a signal of active caspase-3 in these early time frames. Published literature suggests that this staining is rather feasible at 72 h of permanent LAD ligation [35].

Hereby, we detected TUNEL positive cells in the infarct area, underlining the ongoing apoptosis. Interestingly, there was no decline regarding the time course. The TUNEL staining remained constant from approximately 4 h to 48 h. This is in contrast to the decline in autoradiography and PET imaging. However, the TUNEL staining is not directly associated with a change in membrane potential and acidification, but mainly by detecting DNA strand breaks. The different biochemical properties of the TUNEL staining and [^18^F]ML-10 could explain different dynamics. It seems that [^18^F]ML-10 precedes the TUNEL staining and fades over time. It was previously shown that *in vitro* uptake of [^18^F]ML-10 depends on the several characteristic features (e.g., intact, depolarized, acidic and phosphatidylserine-exposing plasma membrane of apoptotic cells [17]), while TUNEL staining rather detects later stages in this process instead. This could represent a technical limitation and might explain the differences. Of note, the histological analysis were performed in the infarct hearts after PET imaging and correct co-localization of TUNEL staining was validated ex-vivo by identifying the infarct area distal to the LAD ligature landmark.

Ma et al. also performed experiments on myocardial infarction in rats [31], demonstrating the decline of TUNEL signal after three to five days. Further, the TUNEL method does not exclusively detect apoptosis but also non-specific DNA degradation [34, 36]. We further could not detect a significant number of apoptotic cells in the remote cardiac tissue. Due to a previous report that differentiated apoptosis and necrosis, we did not further explore features of necrosis in our study [17, 19, 31].

[^3^H]ML-10 was primarily described for selective incorporation into apoptotic cells and not in viable or necrotic cells due to membrane potential and acidification changes. Interestingly, the uptake of [^3^H]ML-10 is lost upon membrane disruption, which on the other hand, is a hallmark of necrosis [17]. This could further explain the decline in [^18^F]ML-10 over time and could offer a conserved time frame for heart tissue at risk. This study did not evaluate the time-activity decline in [^18^F]ML-10 in the autoradiography, which could interfere with the TBR. However, decay correction was performed in the PET experiments.

In this study of myocardial infarction performed by surgical LAD ligation, the operation itself leads to a severe injury by thoracotomy. Detection of cardiac [^18^F]ML-10 in PET imaging could have interfered. This limitation was also observed in a previous study involving myocardial infarction in rats [31]. The accumulation of [^18^F]ML-10 in the lung could be induced by orotracheal intubation and mechanical ventilation, which demonstrates a technical limitation of this work. Future studies are warranted to evaluate the [^18^F]ML-10 accumulation in larger animals, such as pigs, and thereby providing more insight in its feasibility.

Regarding the usage of [^18^F]FDG, factors such as blood flow, substrate availability, hormonal status, insulin sensitivity, and inflammation could potentially interfere with cardiac uptake [37, 38].

Notably, besides the search for the most robust and accurate marker of apoptosis, besides Annexin V as illustrated in the introduction, ongoing research on cardiac hypoxia (reviewed in [39]), or hypoxia identifying [^18^F]fluoromisonidazole (FMISO) [40] or the evaluation of the oxidative metabolism by [^11^C]acetate [41, 42] could be suitable approaches.

## Conclusion

Collectively, this is the first study indicating that [^18^F]ML-10 could be a tracer for imaging apoptosis after permanent LAD ligation in mice. We confirmed the accumulation by a multimodal approach, including autoradiography, *In vivo* small-animal PET imaging, and TUNEL staining of infarcted hearts. Our results support the notion that [^18^F]ML-10 could provide a novel approach for the quantification of apoptosis after myocardial infarction. Further investigations, including in large animal models and, e.g., the ischemia–reperfusion model, could provide further insight into the feasibility and dynamics of [^18^F]ML-10 in the heart.

## Supplementary Information

Below is the link to the electronic supplementary material.

Supplementary file1 (DOCX 28 KB)

## Data Availability

Data are available under reasonable request to the corresponding author.

## Abbreviations

ID/g max: Maximum injected dose per gram
[^18^F]FDG: 2-Deoxy-2-[^18^F]fluoro-D-glucose
[^18^F]ML-10: 2-(5-[^18^F]fluoropentyl)-2-methyl-malonic acid
LAD: Left anterior descending artery
MI: Myocardial infarction
PET: Positron emission tomography
ROI: Region of interest
TBR: Target-to-background ratio
TUNEL: Terminal deoxynucleotidyl transferase dUTP nick end labeling
VOI: Volume of interest
