# Supplementary material for: Detection of cardiac apoptosis by [18F]ML-10 in a mouse model of permanent LAD ligation
Source: Mol Imaging Biol. 2022 Mar 29;24(4):666–74. doi: 10.1007/s11307-022-01718-0 (PMC9296384; doi:10.1007/s11307-022-01718-0)
Supplement: Supplementary file 1 — Supplementary file1 (DOCX 28 KB) [file 11307_2022_1718_MOESM1_ESM.docx]

Supplemental Figure S1:

Figure description:

Quantification of the (%ID/g)max of [^18^F]ML-10 after different time points in sham operated mice. N=5 in total. Data represent mean value.
